# Naturally-occurring DNA fragment termini correlate with methylation at CpG sites in hair and blood plasma cell-free DNA

**DOI:** 10.1186/s12864-025-12459-z

**Published:** 2026-02-20

**Authors:** Samuel Sacco, Joshua D. Kapp, Remy Nguyen, Isha S. Rao, Richard E. Green

**Affiliations:** Department of Biomolecular Engineering, University of California, Santa Cruz, Santa Cruz, CA USA

**Keywords:** Fragmentomics, Cell-free DNA, Hair, Methylation, Forensics, Ancient DNA, Blood plasma, CpG, Epigenetics, Keratinization

## Abstract

**Background:**

CpG methylation is an important epigenetic regulator in growth, development, and disease and a biomarker of age. It was recently shown that CpG methylation influences nuclease activity in the natural process that generates blood plasma cell-free DNA fragments. This results in a correlation between fragmentation sites and methylation that can be used to estimate methylation levels at CpG sites.

**Results:**

We find methylation-dependent fragmentation is also present in the process that generates the cell-free DNA fragments found in hair shafts and urine. Analysis of DNA fragments that includes accurate 5’ and 3’ termini and local sequence context improves modelling of CpG methylation from sequence data.

**Conclusions:**

We demonstrate the existence of methylation-sensitive DNA fragmentation in rootless hair. We develop a model for estimating CpG methylation using both 5-prime and 3-prime native termini of these naturally occurring DNA fragments. This approach enables genotype and epigenetic inference from that same input data. It is applicable for samples that are prevalent in the field of liquid biopsy, forensics and ancient DNA and likely in other keratinized tissues where nuclease digestion of DNA occurs as part of regular development.

**Supplementary Information:**

The online version contains supplementary material available at 10.1186/s12864-025-12459-z.

## Introduction

Cytosine methylation at CpG dinucleotides regulates gene expression in growth, development, and tissue homeostasis. Radical alterations of cytosine methylation is associated with cancerous transformation [1]. Assays that measure CpG methylation in circulating blood plasma cell-free DNA (cfDNA) form the basis of several non-invasive “liquid-biopsy” approaches for detecting tumor presence [2–4] and tissue of origin [5, 6].

CpG methylation has also been shown to vary characteristically across tissue types with environmental exposures [7] and chronological age [8] making it a potentially useful proxy in forensic analysis [9] and ancient DNA [10]. However, the limited and degraded nature of DNA found in cfDNA samples used in liquid biopsy [11] and forensics [12] pose challenges to accurate and cost effective estimation of CpG methylation.

The most-commonly employed method to estimate CpG methylation involves bisulfite conversion of unmethylated cytosines in a DNA sample. This bisulfite-conversion process is then followed by array screening or whole genome sequencing [13]. Bisulfite conversion uses sodium bisulfite to deaminate unmethylated cytosines converting them to uracils. Cytosine methylation status is then inferred by observing cytosine to thymine substitutions in subsequent sequencing [14]. Bisulfite treatment can also cause DNA depyrimidination and fragmentation [15, 16], making the technique unsuitable for degraded samples. Several approaches which avoid bisulfite conversion have been developed, including single-molecule sequencing [17, 18], and enzymatic deamination [19]. However, these alternative methylation assays depend on high molecular weight DNA or specialized library preparations, limiting their applicability in clinical, forensic, and research settings.

Recent advances analyzing the cfDNA fragments found in plasma present an alternative approach for assessment of CpG methylation in data generated by common library preparations [20, 21]. Zhou et al. showed that the in vivo process of nuclease digestion that generates cfDNA fragments is itself at least partially dependent on cytosine methylation. Thus, cytosine methylation generates characteristic patterns in the endpoints of each cfDNA molecule relative to nearby CpG sites, which can be observed in sequencing data as mapped read termini. Specifically, Zhou et al. observed enrichment of 5’ CGN motifs, i.e., fragments whose natural 5’ end starts with a CpG site, and a reduction of 5’ NCG motifs at methylated CpG’s. This pattern is dependent on the activity of *DNAse1L3*, as systemic lupus erythematosus patients with DNASE1L3 deficiency do not show this pattern of methylation-dependent DNA fragmentation [20]. However, the limited observation of 5’ termini (one per sequencing read) requires deep sequencing to accurately estimate this ratio for each of the approximately 28 million CpG sites in the human genome.

In this study, we demonstrate that CpG methylation influences the DNA fragmentation observed in cell-free DNA found in hair shafts and urine in ways similar to those observed in blood plasma cfDNA. Using a single-stranded library protocol that maintains both the native 5’ and 3’ termini of DNA fragments [22], we show that methylation-dependent fragmentation also influences 3’ DNA termini. We show that cytosine methylation - DNA termini associations vary based on the local sequence around a CpG. We present a method for inferring CpG methylation in the DNA from hair at high resolution and accuracy that uses standard DNA sequence data by analyzing native DNA termini and accounting for the local sequence surrounding a CpG.

## Methods

### Previously generated datasets

We downloaded raw methylation array data (idat files) generated in previous studies [23] from the GEO expression database (GSE48472). We used functional normalization implemented in the preprocessFunNorm function of minfi [24] to normalize array methylation beta values between type 1 and type 2 probes. We then calculated the average and standard deviation of beta values at each CpG on the array using the sampled individuals which had hair (*n* = 5) and blood plasma (*n* = 6) data. Lastly, we removed CpG’s with beta values that had high variance between individuals sequenced on the array (SD > 0.05) and for which fewer than 5 termini observations had been made in a 12 bp window. We analyzed sequencing data from hair shafts from a previous study [25] that includes DNA sequence data from 80 hairs from 50 individuals. We pre-processed and aligned the raw data as described in that study and combined BAM files from all of the sequenced individuals into one large BAM to achieve approximately 218X coverage.

### Blood plasma and urine CfDNA data generation

We purchased six human plasma samples in Streck tubes from CGT Global (Folsom, CA) and collected five urine samples from anonymous volunteers under an IRB approved protocol (UCSC HS-FY2025-37). For urine collection, participants picked up and dropped off a sampling kit containing instructions and a Zymo Research Urine Collection Kit (D3062). Participants were requested to donate approximately 100 mL of urine into a collection cup and then combine the provided preservation reagent with the urine sample.

We extracted cfDNA and prepared single-stranded DNA libraries from both the plasma and urine samples. First, we extracted and isolated DNA from 4 mL of plasma or 4 mL of urine using the MagMax Cell-Free DNA Isolation Kit (Applied Biosystems) and quantified the extracts using a Qubit 1X dsDNA High Sensitivity Assay Kit (Invitrogen). Next, we prepared two 1 ng input single-stranded DNA libraries for each plasma extract and one library for each urine extract using the library preparation approach presented in Kapp et al. [22] with the modifications described in the supplemental of Nguyen et al. [26]. Following indexing PCR, we purified the libraries with a SPRI ratio of 1.2X prior to DNA quantification with a Qubit 1X dsDNA HS Assay Kit (Invitrogen) and visualization using a Fragment Analyzer High Sensitivity NGS Assay Kit (Agilent).

We submitted the plasma libraries to the Duke Center for Genomic and Computational Biology for sequencing on a NovaSeq X Plus for one 2 × 150 lane. We sequenced the urine libraries in-house at the University of California, Santa Cruz Ancient and Degraded DNA Processing Center on an Illumina NextSeq 2000 using a 100 cycle 400 M read output kit.

Paired-end raw reads from each plasma sequencing library were adapter trimmed and merged into single reads using SeqPrep. Only merged reads with a minimum length of 30 bp were retained for downstream analysis. We then aligned the merged reads to the GRCh38 human reference with bwa aln, using a seed length (-l) value of 24, followed by bwa samse. The resulting alignment files were next processed with Picard’s CleanSam and MarkDuplicates, and single-library files from each sample were combined into one BAM file using samtools merge for downstream analysis. We uploaded anonymized versions of the six individual bam files to SRA, which were anonymized using BAMboozle v0.5.0 set to strict mode [27]. Final coverage statistics for each of the generated libraries are available in Supplemental Table 1.

Similarly, we processed the sequencing data from the urine libraries with SeqPrep. Raw read pairs were first adapter-trimmed and merged into single reads. Then, we aligned merged reads that are at least 30 bp in length to the GRCh38 human reference using bwa aln, followed by bwa samse. Finally, duplicate reads were removed with samtools rmdup (with -s option). Coverage statistics of the 5 urine samples are shown in Supplemental Table 2.

### Generating termini occurrence counts

**Fig. 1:**
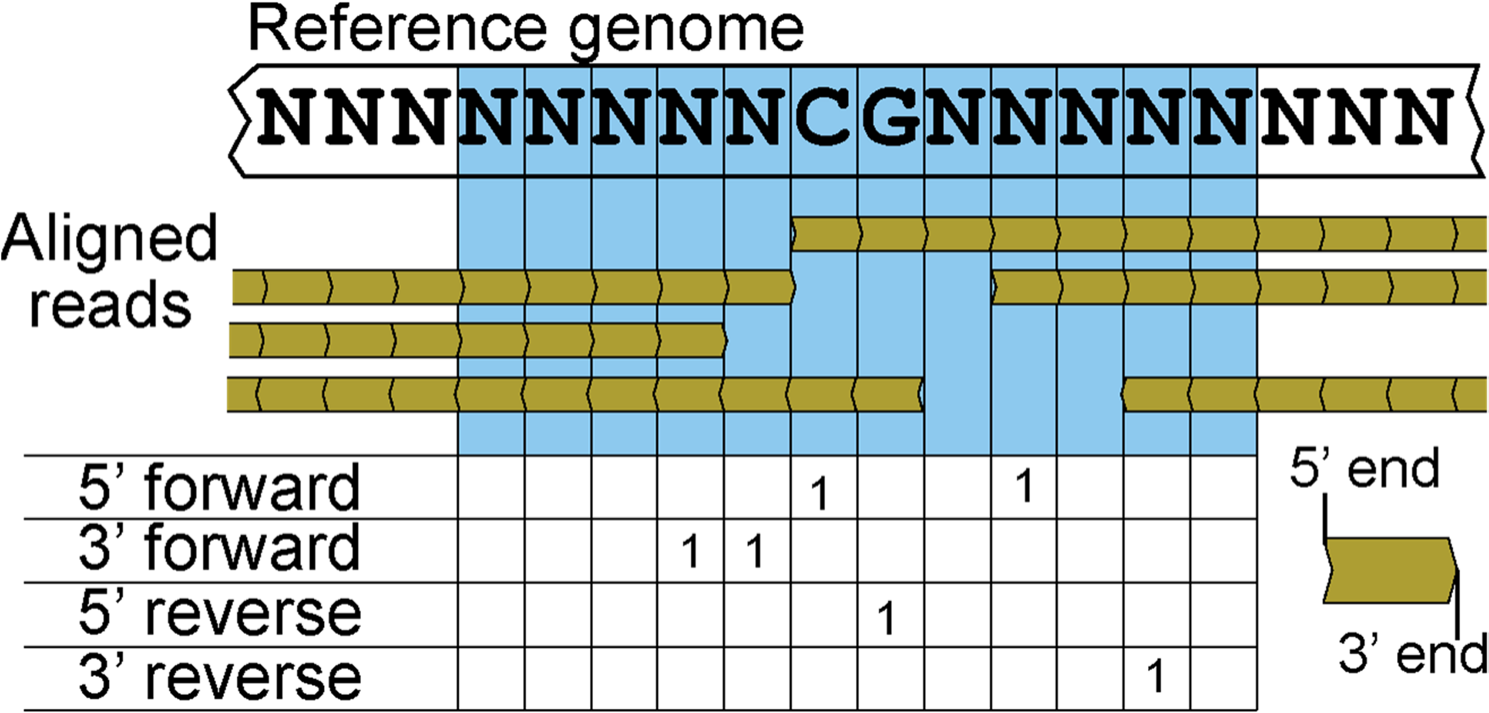
Example of counting termini at a given CpG site in the genome. In blue is the 12 bp window centered on the CpG of interest. Mapped reads are shown in yellow, with a table of the relative counts of aligned termini shown below

For each CpG in the genome, and in each of the three tissue types, we generated counts of where 5’ and 3’ termini occurred in a 12 bp window centered around each genomic CpG (Fig. 1). Within these windows we define position 0 as the position of the cytosine in the CpG dinucleotide and position 1 as the position of the guanine nucleotide on the forward strand. For aligned reads from each tissue, we used samtools mpileup [28] with the following switches (-q 30 -Q 25 -a) to generate a pileup file that had termini occurrences around each 12 bp window (specified with -l using a bed file of CpG windows). We parsed the resulting pileup file using a custom python script which identified CpG windows based on the presence of a “CG” dinucleotide and generated termini occurrence counts from the observations of “$” and “^” characters in the pileup field. We also generated coverage normalized termini counts by dividing the raw counts by the overall coverage at each position. We generate counts of termini solely based on the position of read starts and ends ignoring the sequence of the reads post-alignment, which avoids the higher error rates present at read ends. When parsing with this approach, a 3’ termini observance of the last mapped 3’ base on a read occurs one base pair upstream of the physical cleavage position, whereas for 5’ the cleavage occurs at the first mapped base.

### Termini proportion

Following previous studies [20, 29] we calculated average termini proportions in a 12 bp wide window around each CpG. First, termini observed on the forward and reverse strand were merged in the 5’ to 3’ direction. Then, we calculated the termini proportion as the number of observations of each specific termini type (5’ or 3’) corresponding to cleavage of the DNA strand at a specific position over the total depth of coverage at that position. For analysis of cleavage at hyper and hypomethylated CpG’s we classified CpG’s as hypermethylated if they had an average beta value greater than 0.7 in the array data corresponding to the tissue of interest, i.e. hair follicle array data for hair shaft DNA and blood plasma array data for blood plasma. We classified CpG’s as hypomethylated if they had an array beta value less than 0.3.

### CG/NCG ratio

We calculated CG/NCG ratios by taking the number of 5’ and 3’ termini which corresponded to fragmentation events which generate 5’-CG end motifs and dividing them by the number of termini which corresponded to fragmentation which would generate 5’-NCG motifs. We performed this calculation for rootless hair samples collected from 10 individuals, blood plasma cfDNA collected from 6 individuals, and urine cfDNA collected from 5 individuals. We calculated CG/NCG ratios across all CpG’s present on the GRCh38 human reference genome as well as at CpG’s designed as being present in Alu elements and CpG islands using the repeats and CpG island coordinates obtained from the UCSC table browser [30]. We generated box plots from the distribution of CG/NCG ratios in different individuals and performed Wilcox rank sum tests of significance using the ranksums function implemented in the scipy package [31].

### Termini correlations

We calculated the Pearson correlation coefficient of coverage-normalized termini occurrences obtained from hair shaft and blood plasma cfDNA with the average beta value at each CpG taken from bisulfite array data previously generated [23]. We used hair follicle array data to calculate correlations between termini observed in hair shaft DNA and blood plasma array data to calculate correlations between termini observed in blood plasma cfDNA. To reduce the impact of CpG’s with low termini observances due to low coverage on the correlation calculations we only considered positions around each CpG which were covered by more than 50 reads. This coverage threshold filtered out 6.9% of the total considered sites in hair and 6.3% of sites in plasma.

### Logistic regression modeling

Using coverage normalized occurrences of 5’ and 3’ termini at each position within the 12 bp window surrounding each CpG as features, we trained a logistic regression model using 70% of filtered CpG’s and evaluated model performance on the remaining 30% of CpG’s. We tested both using a single model for the entire dataset and training and testing models separately for each 4mer sequence context. For the 4mer specific models we merged each 4mer with its reverse complement (as these were observed to have the same termini correlation profile). All logistic regression models were implemented in R version 4.4.0. To perform the logistic regression we used R’s generalized linear model implementation with a binomial random component and a logit link to output methylation probabilities between 0 and 1 corresponding to the predicted array beta value of the CpG.

### Downsampling termini observations

We simulated the effect of lower coverage and fewer termini observations on model performance by performing binomial downsampling of termini observances in our original dataset. All counts of observed termini and the coverage at each position in both the train and test dataset underwent binomial resampling in which *n* draws (the number of termini observations at a given position or the coverage at a given position) were performed with a probability *p* corresponding to the downsampling proportion. After downsampling, downsampled termini counts were renormalized using downsampled coverages. The average number of 5’ and 3’ termini observed at each CpG window was then calculated for each downsampled dataset, and model training and testing was performed as described above.

## Results

### CpG methylation dependent fragmentation patterns in hair and plasma CfDNA

**Fig. 2:**
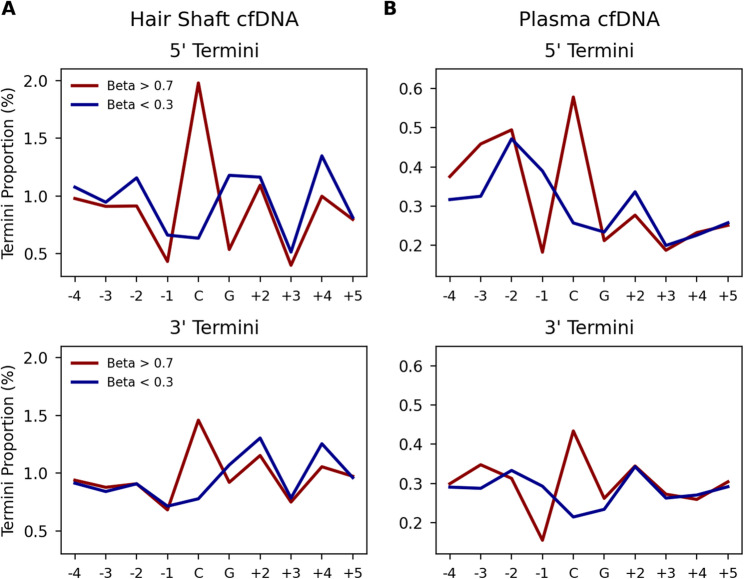
Occurrences of 5’ (top) and 3’ (bottom) molecular termini across a 10 bp window centered around each CpG for DNA isolated from hair shaft (**A**) and blood plasma (**B**). CpG’s binned by array methylation beta value before the proportion of termini was calculated with hypermethylated (beta > 0.7) shown in red and hypomethylated (beta < 0.3) shown in blue

To investigate the relationship between DNA fragmentation points and cytosine methylation, we used previously generated sequence data from 80 rootless hairs [25] and generated data from six blood plasma samples and five urine samples. All libraries were prepared using a single-stranded DNA library prep that maintains the native ends of the input DNA fragments [22]. We then mapped the sequence data to the human reference genome and used these mapping coordinates to determine the sequence context around each fragmentation position, i.e., the first mapped base and the last mapped base (Fig. 2.).

We compared the DNA sequence fragment data to published data from the 450 K Illumina Infinium Methylation array data of hair follicles (for fragmentation data observed in hair shaft cfDNA) or blood plasma (for fragmentation data observed in plasma cfDNA) [23]. For each position within a 10 base-pair window around each CpG we compared the observed proportion of molecular termini at each position with the array methylation proportion (Beta Value). For this analysis, we compared only CpGs with Beta Values of larger than 0.7 (hypermethylated) or less than 0.3 (hypomethylated) (Fig. 2a).

In hair shaft cfDNA at 128,884 hypermethylated CpG’s we observed an average 5’ termini proportion of 1.98% at the C position compared to a 0.63% 5’ termini proportion in 144,634 hypomethylated CpG’s (3.14x fold increase). In plasma cfDNA we observe an average 5’ termini proportion of 0.58% across 52,232 hypermethylated CpG’s and an average 5’ termini proportion of 0.26% across 110,403 hypomethylated CpG’s (2.23x fold increase). We observed similar differences in the proportion of observed 3’ termini at the C position, though the fold increase between hyper and hypomethylated CpG’s is smaller in both hair (1.89x fold increase) and plasma (2.04x fold increase). Other notable differences between termini proportions in hypermethylated and hypomethylated CpG’s included the − 1 position in plasma (2.2x fold decrease 5’ and 1.93x decrease 3’ ) and the G position in hair (2.18x decrease in hyper relative to hypomethylated). These fold increases and decreases between hyper and hypo methylated CpGs were statistically significant (*p* < 0.001) when using a Wilcoxon-rank sum test. We note that each aligned DNA fragment has single 5’ and 3’ terminal positions. Thus, the termini proportion at each position necessarily derives from independent sequence fragments, i.e., these results are additive for inferring methylation status from termini end-points.

### Correlations of 5’ and 3’ termini with methylation globally and at specific sequence contexts

**Fig. 3:**
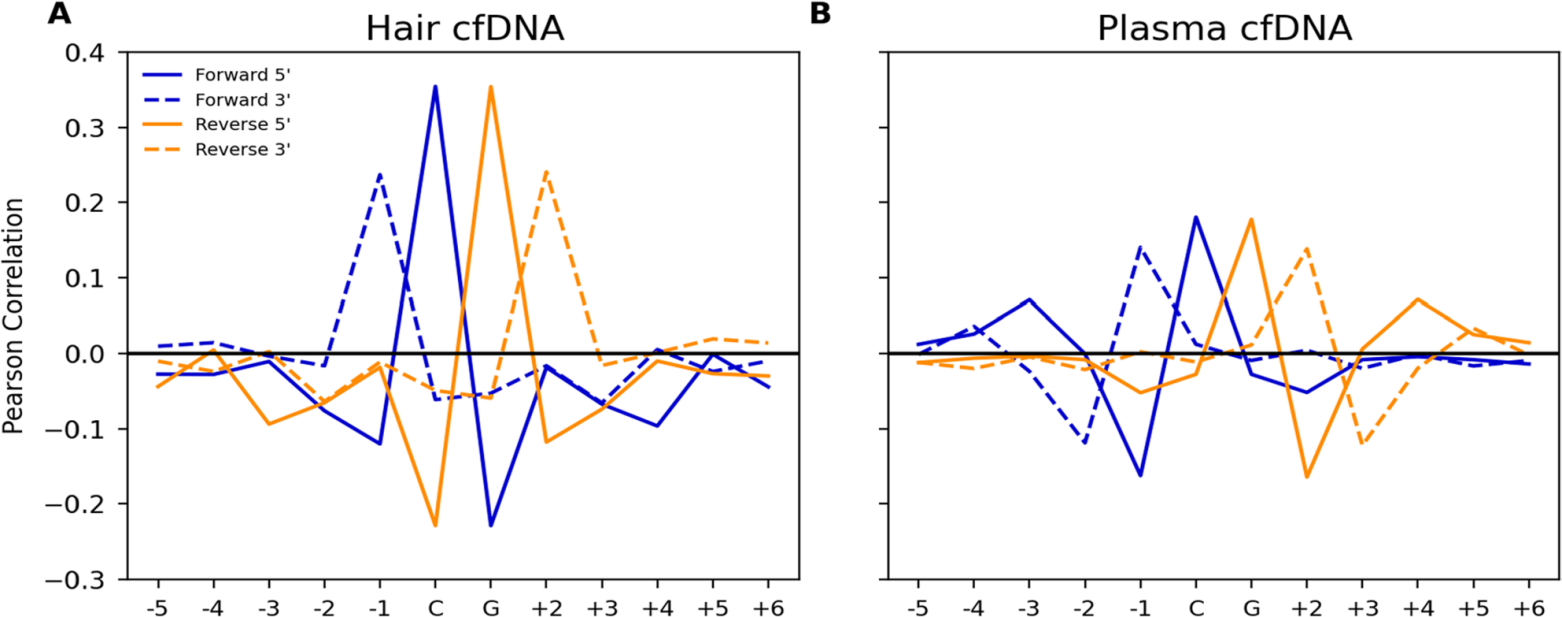
Pearson correlations of coverage normalized occurrences of 5’ and 3’ termini in a 12 bp window around each CpG. Correlations were calculated against the beta value of that CpG in bisulfite array data from hair follicle or blood plasma. Correlations were generated for hair shaft cfDNA (**A**) and plasma cfDNA (**B**)

We calculated Pearson correlation coefficients between the array methylation proportion (Beta Value) of each CpG and the coverage normalized occurrences of 5’ and 3’ termini on the forward and reverse strands at each position within a 12 base-pair window around each CpG (Fig. 3). Beta values used in the correlation calculations are the average Beta Value from hair follicle array data taken from 5 individuals (Fig. 3a) and average Beta Values taken from blood plasma from 6 individuals (Fig. 3b). We calculated the correlations using a subset of the 450 K CpG’s for which at least 50 reads cover the position of interest (93.1% of 5.2 million sites in hair and 93.6% of ~ 4.8 million sites in plasma).

We observed a high level of symmetry in correlated termini between the forward and reverse strands in both hair shaft DNA and blood plasma cfDNA. In hair and blood plasma cfDNA 5’ and 3’ termini that correspond to a fragmentation of the DNA strand at the C position at the CpG site are the most positively correlated with methylation status. In blood plasma cfDNA, 5’ and 3’ termini corresponding to fragmentation one base pair upstream of the C position in the CpG are the most negatively correlated. In contrast, the most negatively correlated termini in hair cfDNA are exclusively 5’ and correspond to fragmentation at the G position.

In both hair and plasma cfDNA the magnitude of the correlations for 5’ termini are larger than 3’ termini even when these termini correspond to the same fragmentation location along the DNA strand. This difference in correlations between 5’ and 3’ termini is larger in hair where 5’ termini corresponding to fragmentation just upstream of the C position have correlations on the forward and reverse strand of *r* = 0.355 and *r* = 0.354 and the 3’ termini corresponding to this same fragmentation have a correlation of *r* = 0.236 and *r* = 0.241, a ~ 33% decrease in correlation between 5’ and 3’. In comparison, in plasma cfDNA 5’ termini associated with fragmentation just upstream of the C position have correlations on the forward and reverse strand of *r* = 0.180 and *r* = 0.177 whereas 3’ termini have correlations of *r* = 0.140 and *r* = 0.138, a ~ 22% decrease between 5’ and 3’.

**Fig. 4:**
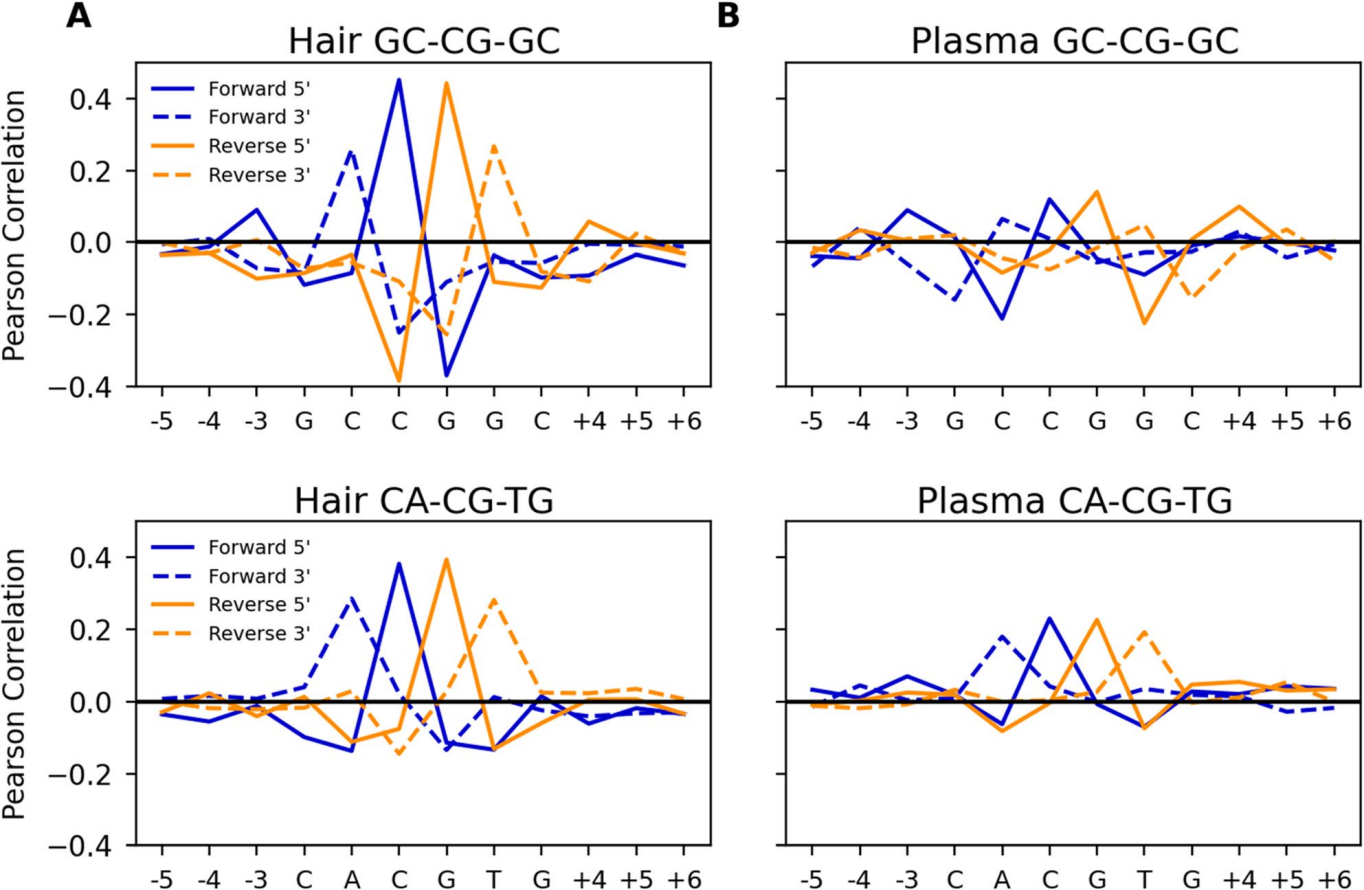
Example termini-methylation correlation profiles at two 2 different 4mer sequence contexts in hair (**A**) and plasma (**B**)

We further explored the correlation between fragmentation termini and methylation level by examining it considering the local sequence context around CpG sites. We classified each CpG by the two base pairs preceding and following it, merging contexts which were the reverse complement of one another. In this way, each CpG is put into one of 136 base context categories. All CpG sites in the same category were then examined together. We found that many of the strongest correlations were present in most sequence contexts, but with variations. Figure 4a and b show an example comparison between the 4mer contexts GC**CG**GC and CA**CG**TG in hair and plasma. We found that many patterns of termini/methylation correlations vary in both blood plasma cfDNA and hair cfDNA depending on the surrounding 4mer sequence context.

### Fragmentation-estimated CpG methylation in genome regions known to be Hyper- and hypomethylated

The human genome contains the remnants of about one million mobile Alu elements. As a class, these are known to be hypermethylated [32]. This methylation is thought to be involved with inactivating their transcription and further mobilization [33, 34]. Alternatively, CpG islands are genomic regions whose methylation status controls activation of nearby genes. Although these are dynamically regulated, as a class, they tend to be hypomethylated [35]. To further validate the correlation between cytosine methylation and cfDNA DNA fragmentation in hair, blood plasma, and urine, we analyzed DNA fragmentation patterns at CpG sites found in these genomic elements (Alu and CpG Island) and compared to genome-wide averages.

**Fig. 5:**
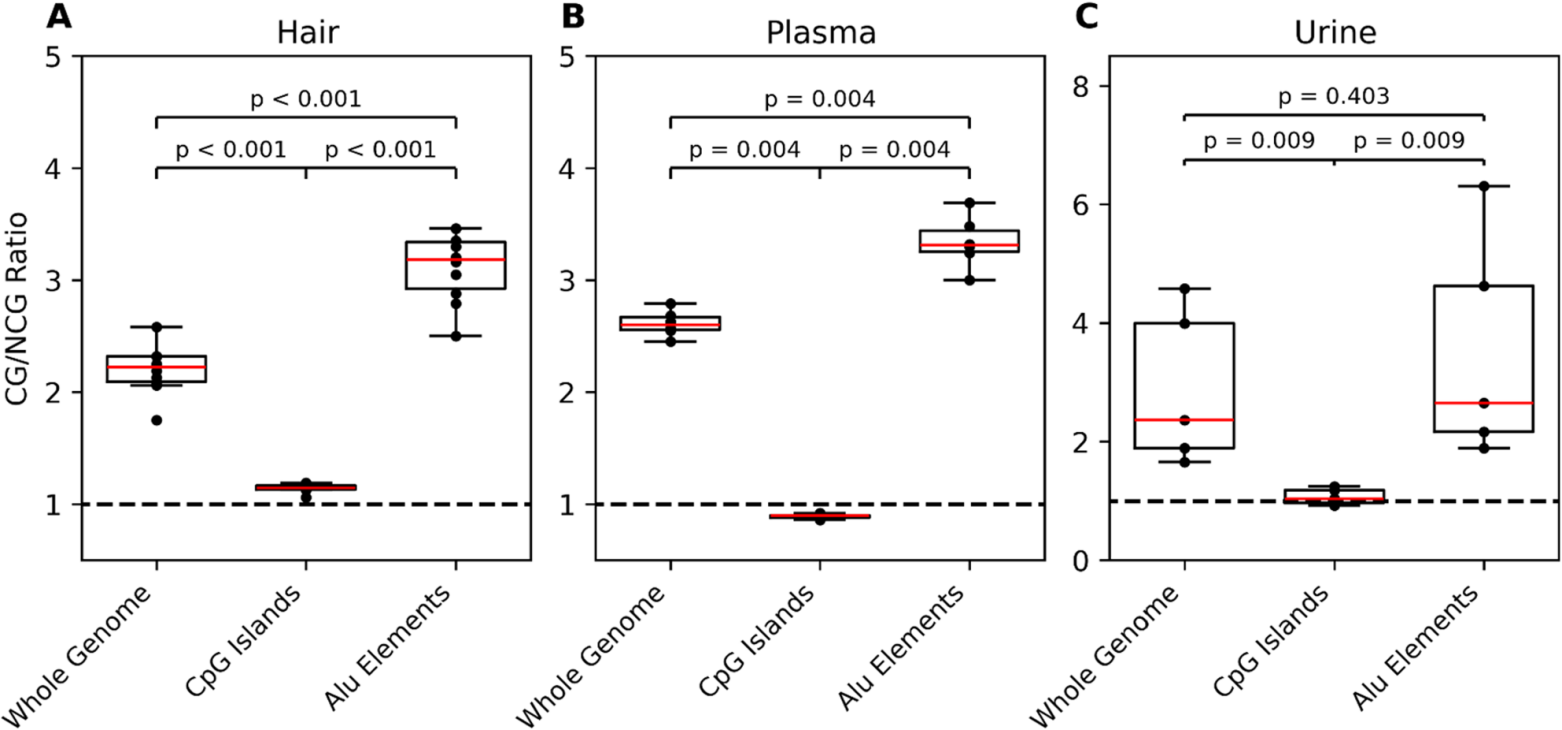
Distribution of CG/NCG ratios calculated using 5’ and 3’ termini among individuals sampled from plasma (*n* = 6), urine (*n* = 5), and hair (*n* = 10) across the genome and at CpG islands and Alu elements. Reported p values are the results of a Wilcoxon-Rank Sum test between groups

For this analysis, we calculated the CG/NCG ratio using both 5’ and 3’ termini. In both hair and plasma the CG/NCG ratio at Alu elements is significantly higher than across the genome (hair: *p* < 0.001, plasma *p* < 0.01) or at CpG islands (hair: *p* < 0.001, plasma *p* < 0.01) (Fig. 5). This pattern also holds true in DNA fragments from urine, but only when comparing the CG/NCG ratio at the different genomic elements within the same individual (Supplemental Figure S1). We also calculated the CG/NCG ratio in each tissue at each element using only 5’ (Supplemental Figure S2) and 3’ termini (Supplemental Figure S3). Across the entire genome and at Alu sites CG/NCG ratios are lower when calculated using 3’ termini as opposed to 5’ termini in hair and plasma.

### Modeling CpG beta values in hair shaft CfDNA from termini

Given the correlation between DNA fragment termini and degree of cytosine methylation and that the nuances of this relation are dependent on the sequence context surrounding the CpG site, we set out to develop a quantitative predictor of methylation status based on the observed DNA fragment termini in proximity to a CpG site. To train the predictor, we performed logistic regression, estimating each CpG’s average beta value from coverage-normalized observances of termini within the 12 bp window centered at each CpG. We used the available data at 70% of the CpG sites to train each model and then tested the model fit on the 30% of the data left out of the training set. We tested using a single logistic regression model for all CpGs versus training 256 individual logistic regression models for each 4mer sequence context (merging data from each 4mer with its reverse complement).

**Fig. 6:**
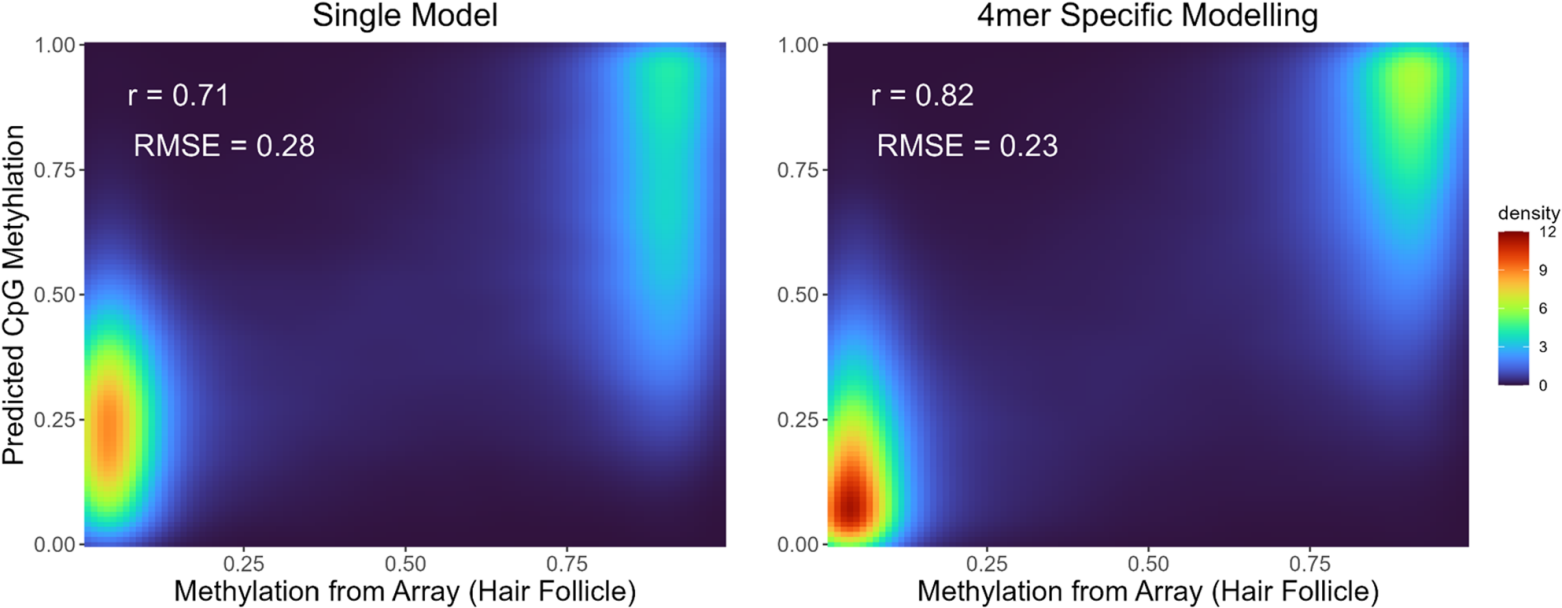
Density heatmap of predicted CpG methylation from termini observed in hair shaft cfDNA vs. CpG methylation on array when using a single logistic regression model (A) and separate models for each 4mer sequence context. Calculated density corresponds to the number of CpG’s on the array with the given predicted/array estimated methylation values

We observed that using separate models for each 4mer improved overall prediction accuracy (*r* = 0.82) versus a single model trained over all CpG sites (*r* = 0.71) (Fig. 6). The model trained on hair DNA fragment data achieved higher overall accuracy compared to the model trained with blood plasma cfDNA data using either modeling strategy, though accuracy was also improved in plasma when using separate models (*r* = 0.52) (Supplemental Figure S4 and Supplemental Figure S5).

### 5’ and 3’ termini impact model performance and convey information about CpG methylation status in hair

Standard DNA library preps often perform end-repair before adapter ligation. This end-repair involves T4 polymerase treatment that fills in 5’ overhangs and removes 3’ overhangs by exonuclease activity. The end-result of this treatment retains only the native 5’ termini of input DNA fragments. We sought to quantify the additional information provided by capturing both the native 5’ and 3’ termini as done when generating the hair, blood plasma, and urine DNA examined here.

**Fig. 7:**
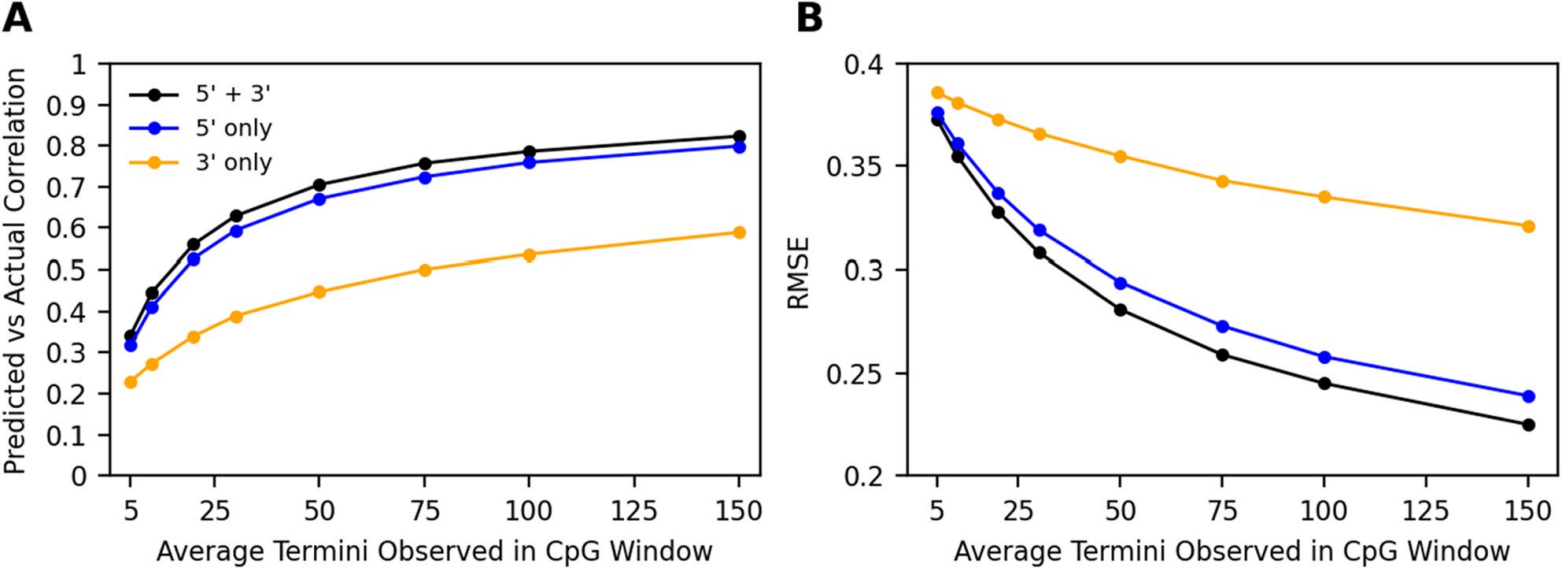
Effect of downsampling termini observances obtained from hair shaft sequencing data on the performance of logistic regression models using both 5’ and 3’ termini as features, 5’ only, and 3’ only. Pearson correlation between predicted and array beta values are shown left (**A**) and RMSE is shown right (**B**)

Using our 4mer specific logistic regression model to predict methylation from termini observed in the hair shaft data, we tested the relationship between model performance and the average number of termini observed in each CpG window when training models using 5’, 3’ and both classes of termini (Fig. 7). We downsampled termini observances at all positions by performing *n* draws of termini observations (number of termini observed at position in the original data) with a probability given by a specified downsampling proportion. We then calculated the average number of termini (both 5’ and 3’) observed per window after downsampling and then retrained and retested models using these downsampled datasets, reporting the correlation between predicted and array CpG beta values and the root mean squared error (RMSE).

Correlations between predicted and array CpG beta values were highest, and RMSE was lowest, at all levels of termini observances for models which used both 5’ and 3’ termini data. Models using 5’ termini performed similarly but slightly worse than 5’ and 3’ models in hair cfDNA with an increase in correlation of 2.9% when 150 termini are observed in the CpG window (5’ *r* = 0.799 and 5’+3’ *r* = 0.823). In plasma cfDNA, we observed a larger increase in model performance when using 5’ + 3’ termini as compared to 5’ only (Supplemental Figure S6), with 30 termini observances per window model correlations having an increase in correlation of 10% (5’ *r* = 0.442 and 5’ + 3’ *r* = 0.491).

## Discussion

Until now, observations of CpG methylation-influenced patterns of DNA fragmentation have been limited to cfDNA isolated from blood plasma [20, 21]. We demonstrate that similar methylation-influenced fragmentation patterns also exist in urine and hair shaft cfDNA. Our results are consistent with models of methylation-influenced fragmentation occurring due to the cutting preference of endonucleases [20], as endonucleases play an essential role in DNA fragmentation during the cornification of keratinocytes [36–38]. In plasma cfDNA, *DNAse1L3* has been identified as a candidate for creating the observed methylation dependent CG/NCG signal [20, 39]. While our analysis does not address which endonucleases generate methylation-influenced fragmentation in hair DNA, possible candidates include endonucleases expressed in keratinocytes such as *DNAse1l2* and *DNAse2* which have been found to be essential to DNA degradation during cornification [37, 38]. Identification of these methylation-influenced nucleases could enable exploration of these patterns in tissues where keratinization or other processes involving regulated digestion of DNA occurs. More broadly, endonucleases involved in the keratinization of tissues such as *DNAse1l2* and *DNAse2* are conserved across vertebrate taxa [40] opening possibilities of observing this pattern in other species.

While methylation dependent fragmentation patterns exist in both hair and plasma, the correlations observed between termini and CpG methylation vary between the two tissues and within tissues by local sequence context. Differences in termini correlations with methylation between hair and plasma, and differences between local sequence contexts within the same tissue, could both be the result of the sequence preferences of tissue-specific endonucleases or differential activity of additional endonucleases and exonucleases after the initial endonucleolytic fragmentation.

Any DNA strand break, created by an endonuclease or non-enzymatically, will generate a single 3’ terminus upstream of the break and a single 5’ terminus downstream of the break. One might expect, then, that both 5’ and 3’ correlations with any fragmentation event will be of equal magnitude. However, we observe that 3’ termini often have lower magnitude correlations with methylation than adjacent 5’ termini, especially for DNA fragments found in hair shafts (Fig. 2). One possibility is the action of 3’ exonucleases at least partially ablating the original fragmentation signal at the 3’ ends of DNA fragments. Consistent with this model is the observation that hair follicles express 3’ exonucleases such as *Trex2* [41, 42]. Increased activity of 3’ exonucleases in hair could also explain the larger decrease in CG/NCG ratio observed in hair cfDNA when calculated using only 3’ termini as opposed to 5’ relative to plasma (Supplemental Figure S4. and Supplemental Figure S5.).

Despite the reduction in methylation signal at 3’ termini, our downsampling analysis demonstrates that both 5’ and 3’ termini can be predictive features of CpG methylation status individually and additively. While models trained exclusively with 5’ termini predict CpG methylation in hair cfDNA at higher accuracy than models trained on only 3’ termini, model performance is highest using both 5’ and 3’ - an expected result since these termini data are independent and both contain information. Higher accuracy modelling of CpG methylation at lower relative sequencing cost can then be achieved by adopting single stranded library preparations which recover native 5’ and 3’ termini when generating data for fragmentomic analysis. Using 3’ termini as features seems especially important in plasma cfDNA, where 3’ termini have correlations nearly as strong as 5’ (Fig. 2B) and 3’ trained models predict methylation status more similarly to 5’ trained models (Supplemental Figure S6).

We observe stronger methylation-influenced fragmentation signals in DNA from hair than in DNA from blood plasma. This is demonstrated most noticeably in the large difference of predictive accuracy between hair shaft models (*r* = 0.82) and plasma models (*r* = 0.52). Hair cfDNA, with average fragment lengths of 30–50 bp [25] is more fragmented than plasma cfDNA which has a modal size of ~ 166 bp [11]. While differential coverage between the two sample types is one contributor to these differences in accuracy, when controlling for the number of termini observed within a CpG window hair cfDNA fragmentation regression models still more accurately predict CpG methylation (Fig. 6. Vs Supplemental Figure S6) - meaning hair cfDNA not only has more termini at a given coverage (due to its more fragmented nature), but that these termini are more informative of methylation status. One explanation could be that during keratinization, DNA is more thoroughly fragmented, as evidenced by the shorter DNA fragment lengths in hair relative to plasma and that the fragmentation process occurs primarily in a methylation-aware fashion. In blood plasma, DNA is less fragmented and perhaps the result of methylation-aware and methylation independent processes as blood plasma cfDNA is known to be fragmented by multiple endonucleases such as *DNASE1L3*, *DFFB* and *DNASEL1* [11, 20].

The results shown in this study have several caveats. First, the plasma (*n* = 6) and urine (*n* = 5) cfDNA datasets had smaller sample sizes and were sequenced at lower coverage than the hair shaft samples (Supplemental Tables 1 and 2). As a result, plasma cfDNA contained fewer termini per CpG window relative to hair when no downsampling was performed, and urine cfDNA lacked sufficient coverage to support direct modeling of cytosine methylation from termini. Regardless, the urine data still showed elevated CG/NCG ratios at CpG’s aggregated across the genome and within Alu elements relative to CpG islands, consistent with methylation-aware fragmentation in this sample type. Second, our downsampling analyses in plasma and hair shaft cfDNA (Fig. 7 and Supplemental Fig. 6) indicate that accurate methylation inference requires observing a sufficient number of termini around each CpG, implying that high-coverage sequencing or the combination of multiple low-coverage samples is necessary to accurately predict methylation with this approach. Third, although fragmentation patterns in hair shaft cfDNA predict hair follicle array methylation accurately (*r* = 0.82), methylation in the shafts likely differs slightly from that of the follicle cells. Direct estimation of methylation within hair shaft cfDNA in future studies could enable more accurate fragmentation-based methylation prediction in this tissue type specifically.

A DNA fragmentation-based estimator of cytosine methylation from hair shaft DNA could allow an epigenetic-based approach to forensic applications given the correlation between environmental variables, such as age, and epigenetic modifications [8, 9]. Similarly, in ancient DNA, fragmentation based reconstruction of CpG methylation in hair, when combined with traditional substitution based approaches [43], could generate higher resolution methylation maps of ancient samples than previously obtainable [44]. Combined with an understanding of the relationship between CpG methylation and environmental variables like stress, age, and disease these methylation maps could provide insight into the environmental contexts that shaped human history and that of other animals beyond what genetic data can tell us [10]. Expanding the range of keratinized tissues with observed methylation-aware DNA fragmentation to horns, hooves, feathers, and other tissues could enable analyses in the emerging field of conservation epigenetics [45, 46]. However, studies establishing the enzymatic mechanisms of methylation-aware fragmentation in hair shaft and other keratinized tissues either through knockouts, as has been done in mice to identify candidate enzymes in blood plasma [20, 47], or other means are needed to inform future fragmentation based modelling strategies and identify candidate tissue types with methylation-sensitive fragmentation.

The fragmentation based models developed here for analysis of cytosine methylation require no special chemical or enzymatic treatment of the DNA sample. The underlying signal is the consequence of the natural fragmentation process that generates these cell-free DNA fragments. Thus, capturing this signal requires only standard DNA library preparation or single-stranded library prep to capture both the 5’ and 3’ components of the signal. This advantage is particularly important for applications such as ancient DNA [48–50] and forensics [51] where samples are limited and irreplaceable. In this way, there is no compromise between the primary goal of the emerging field of SNP-based forensics - analysis of genetic variants - and further methylation analysis. The same data serves two purposes.

## Conclusions

Our results demonstrate the existence of methylation-sensitive fragmentation in cfDNA obtained from rootless hair shafts and urine. These fragmentation patterns enable accurate estimation of CpG methylation status in hair cfDNA at single CpG resolution solely from shotgun sequencing data, when coverage is sufficiently high. We demonstrate that approaches to modelling CpG methylation from observances of DNA termini are improved in hair and blood plasma when considering 5’ and 3’ termini together and further improved in these tissues when accounting for the base context around a given CpG.

## Supplementary Information

Supplementary Material 1.

## Data Availability

The urine cfDNA sequencing data generated in this study are available in dbGaP under accession number phs004104.v1.p1. Data from rootless hairs used in this study is available at dbGap under accession phs002979.v2.p1. Anonymized bam files are available for the six plasma cfDNA samples on SRA under BioProject ID: PRJNA1246997. Python code for parsing termini at CpG sites from pileup files is available at (https://github.com/Paleogenomics/Hair_Fragmentomics).

## Abbreviations

cfDNA: cell-free DNA
RMSE: Root Mean Squared Error
bp: Base Pair
PCR: Polymerase Chain Reaction
