# Supplementary material for: Naturally-occurring DNA fragment termini correlate with methylation at CpG sites in hair and blood plasma cell-free DNA
Source: BMC Genomics. 2026 Feb 20;27:308. doi: 10.1186/s12864-025-12459-z (PMC13023193; doi:10.1186/s12864-025-12459-z)
Supplement: Supplementary file 1 — Supplementary Material 1. [file 12864_2025_12459_MOESM1_ESM.docx]

**Supplemental Material**

**Supplemental Table S1**

| Plasma sample | Library | Merged reads | Unique GRCh38 data (bp) | Depth of coverage |
| --- | --- | --- | --- | --- |
| JK-Plasma-E001 | JK-Plasma-E001-L001 | 256,238,004 | 22,224,000,126 | 16.13 |
|  | JK-Plasma-E001-L002 | 250,988,963 | 26,169,312,674 |  |
| JK-Plasma-E002 | JK-Plasma-E002-L001 | 218,801,344 | 24,186,960,932 | 15.78 |
|  | JK-Plasma-E002-L002 | 219,628,639 | 23,160,132,703 |  |
| JK-Plasma-E003 | JK-Plasma-E003-L001 | 214,546,700 | 25,916,057,893 | 16.75 |
|  | JK-Plasma-E003-L002 | 229,850,983 | 24,334,597,761 |  |
| JK-Plasma-E004 | JK-Plasma-E004-L001 | 229,581,527 | 25,621,024,307 | 16.90 |
|  | JK-Plasma-E004-L002 | 209,314,605 | 25,065,664,077 |  |
| JK-Plasma-E005 | JK-Plasma-E005-L001 | 236,116,159 | 24,503,551,612 | 17.43 |
|  | JK-Plasma-E005-L002 | 198,200,259 | 27,792,990,657 |  |
| JK-Plasma-E006 | JK-Plasma-E006-L001 | 249,121,179 | 27,792,809,181 | 18.68 |
|  | JK-Plasma-E006-L002 | 234,083,902 | 28,260,426,263 |  |

**Table S1**. Sequencing statistics of the 6 tested plasma samples.

**Supplemental Table S2**

| Urine sample | Library | Merged reads | Unique GRCh38 data (bp) | Depth of coverage |
| --- | --- | --- | --- | --- |
| PGL-U001-E001 | PGL-U001-E001-L001 | 136,289,740 | 480,776,984 | 0.160 |
| PGL-U002-E001 | PGL-U002-E001-L001 | 142,712,984 | 538,074,012 | 0.179 |
| PGL-U003-E001 | PGL-U003-E001-L001 | 145,038,884 | 103,693,262 | 0.0346 |
| PGL-U005-E001 | PGL-U005-E001-L001 | 153,329,784 | 87,988,611 | 0.0293 |
| PGL-U007-E001 | PGL-U007-E001-L001 | 144,430,616 | 363,206,208 | 0.121 |

**Table S2**. Sequencing statistics of the 5 tested urine samples.

**Supplemental Figure S1**


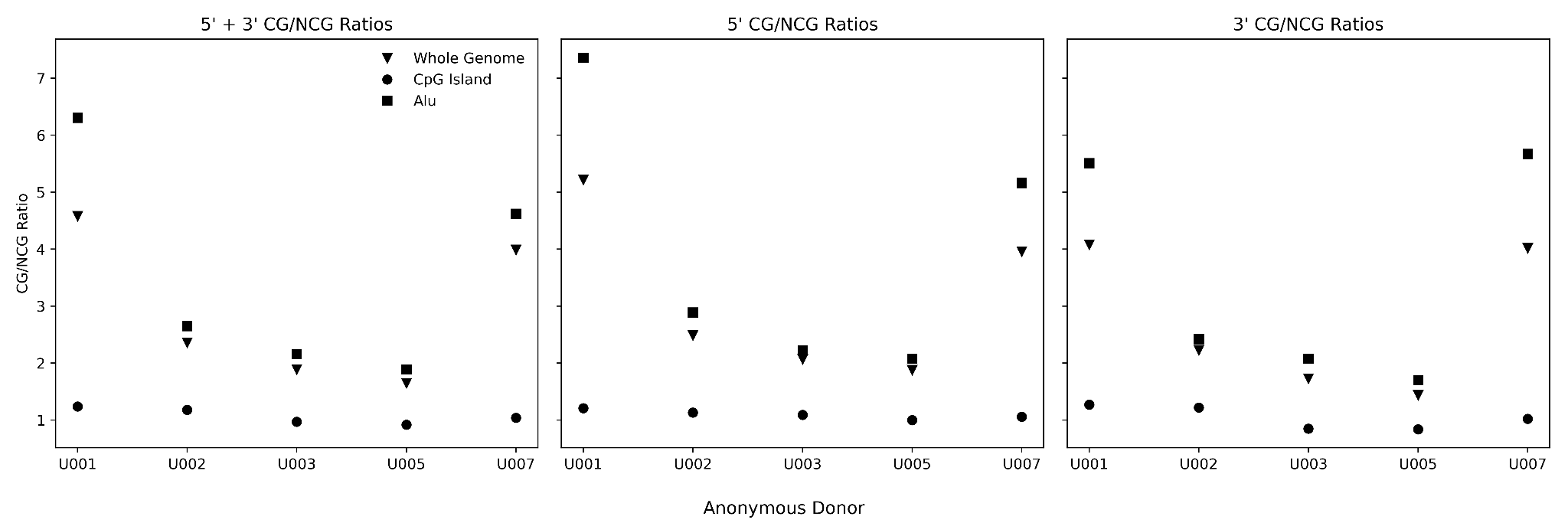


**Fig S1.** Distribution of CG/NCG ratios calculated using different termini between the 5 anonymous urine samples measured across the whole genome, CpG islands, and Alu elements.

**Supplemental Figure S2**


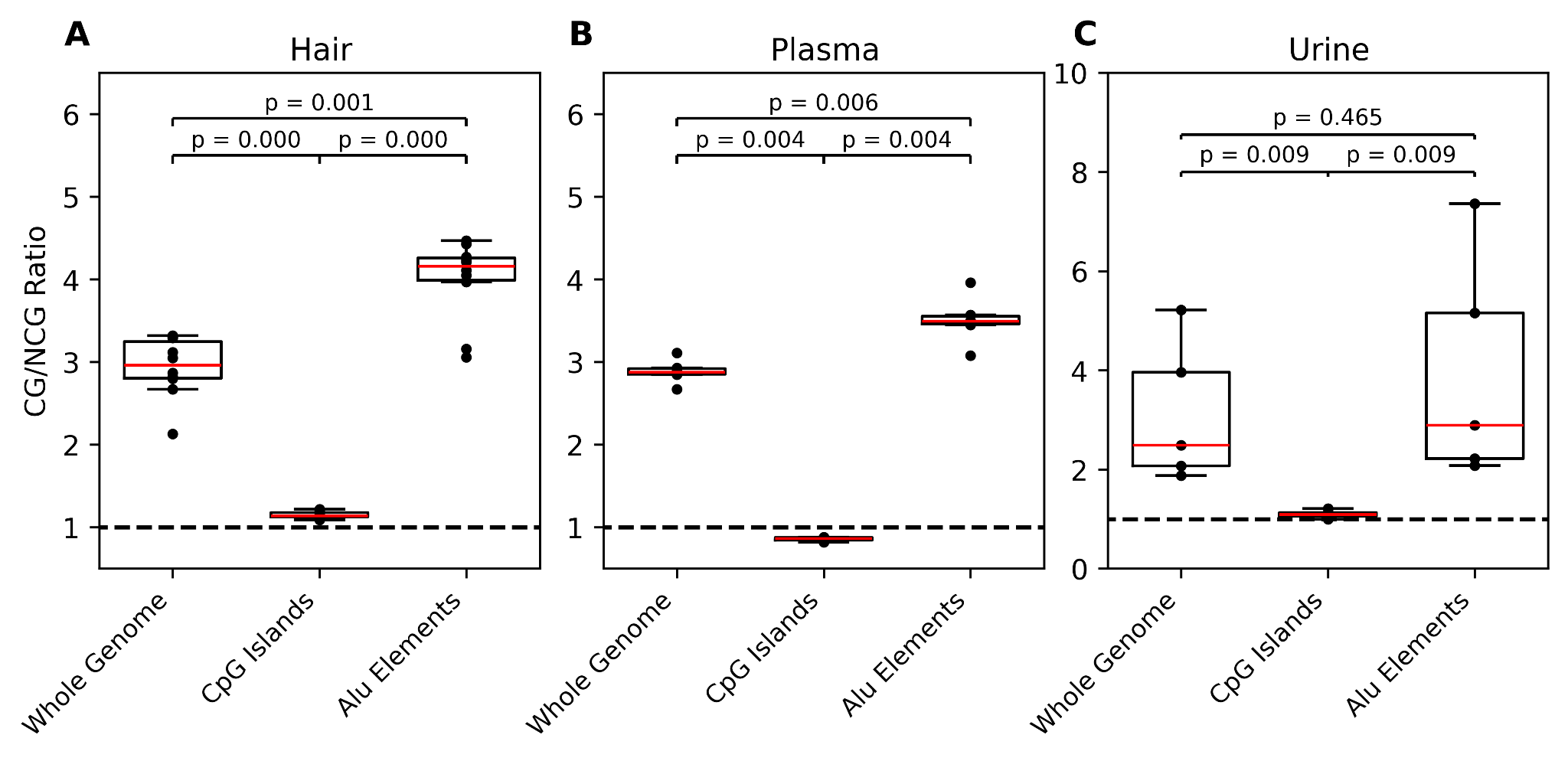


**Fig S2.**  Distribution of CG/NCG ratios calculated using 5’ termini exclusively among individuals sampled from hair (n=10), plasma (n=6), and urine (n=5) across the genome and at CpG islands and Alu elements. Reported p values are the results of a Wilcoxon-Rank Sum test between groups.

**Supplemental Figure S3**


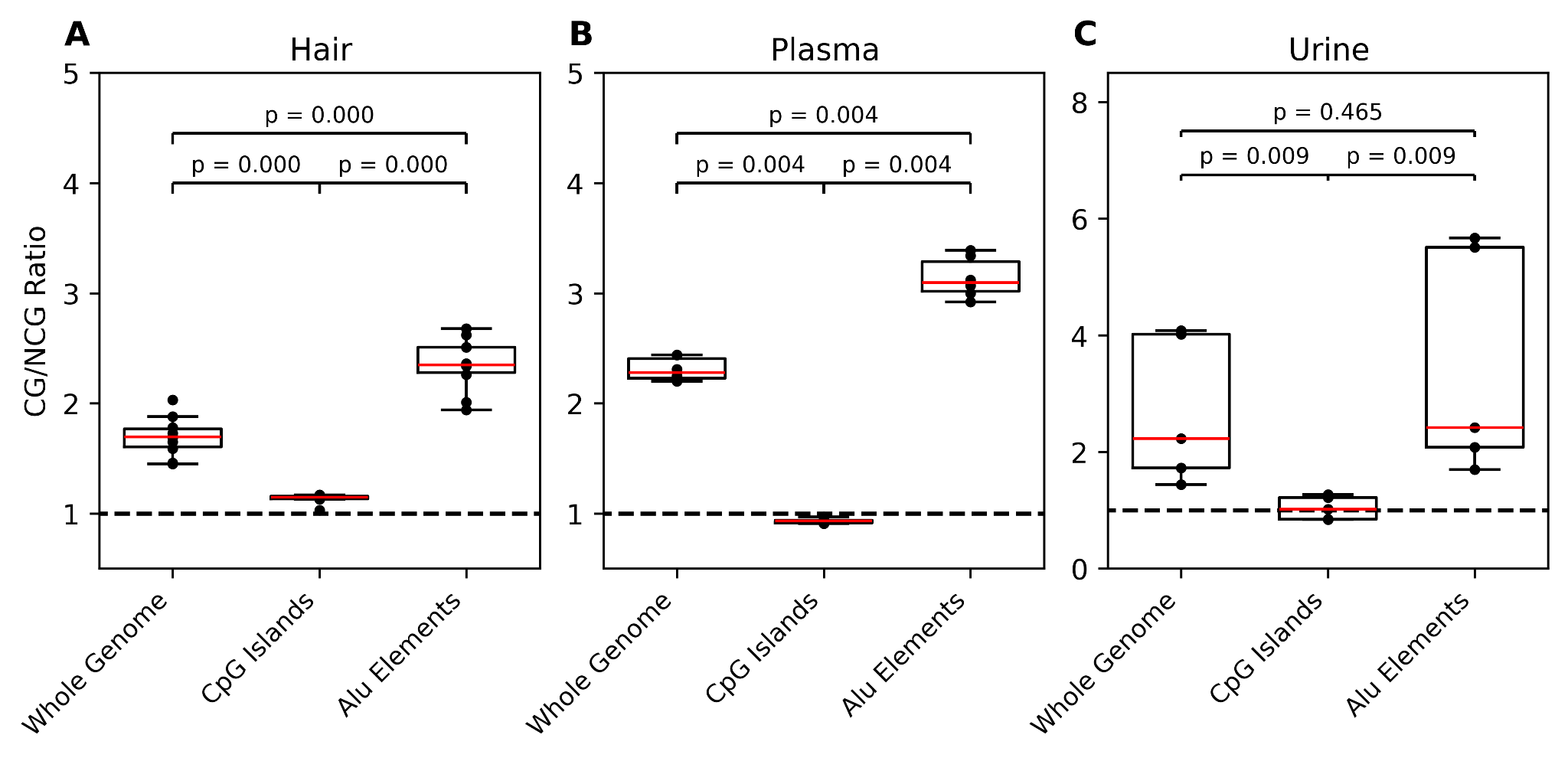


**Fig S3.** Distribution of CG/NCG ratios calculated using 3’ termini exclusively among individuals sampled from hair (n=10), plasma (n=6), and urine (n=5) across the genome and at CpG islands and Alu elements. Reported p values are the results of a Wilcoxon-Rank Sum test between groups.

**Supplemental Figure S4**

**
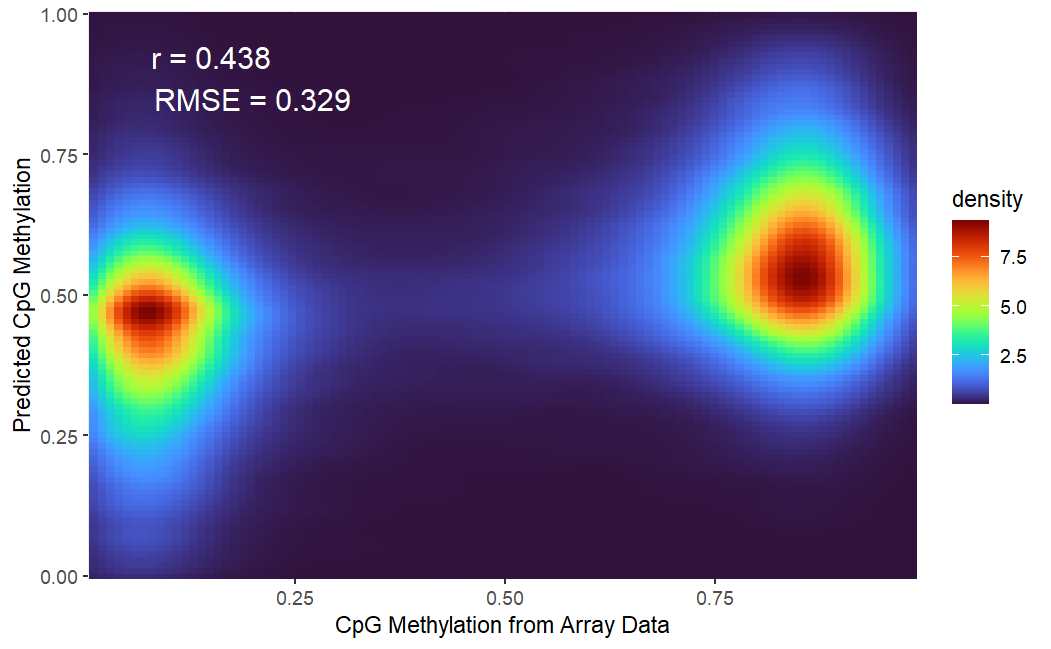
**

**Fig S4**. Density heatmap comparing predicted CpG methylation to observed CpG methylation in blood plasma samples using a single logistic regression model for all CpG’s with termini features.

**Supplemental Figure S5**

**
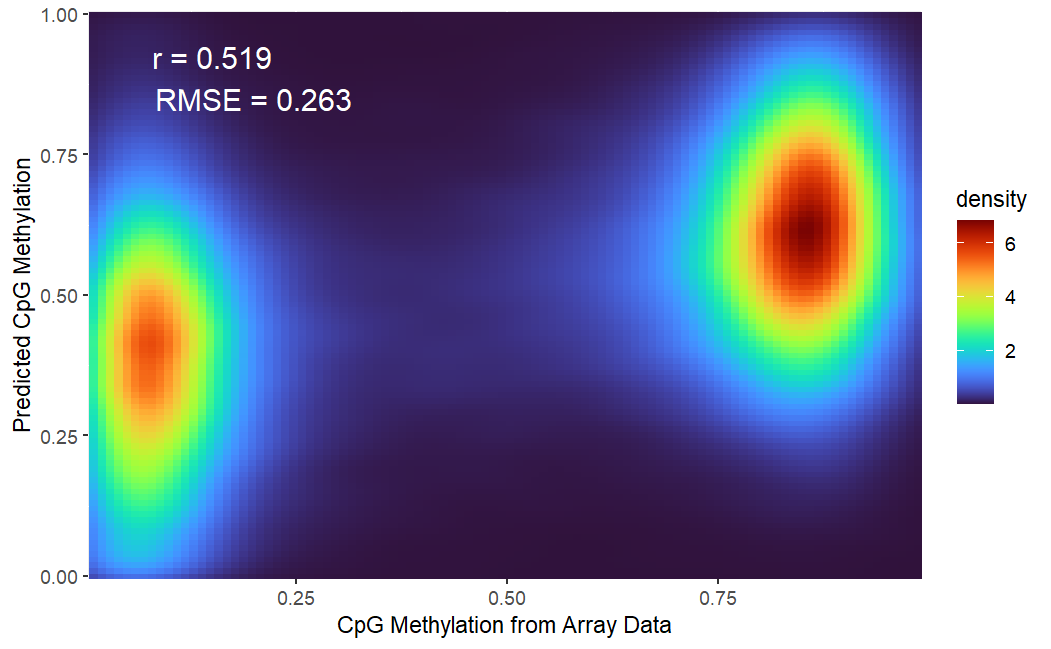
**

**Fig S5**. Density heatmap comparing predicted CpG methylation to observed CpG methylation in blood plasma samples using separately trained logistic regression models for each 4mer sequence context.

**Supplemental Figure S6**


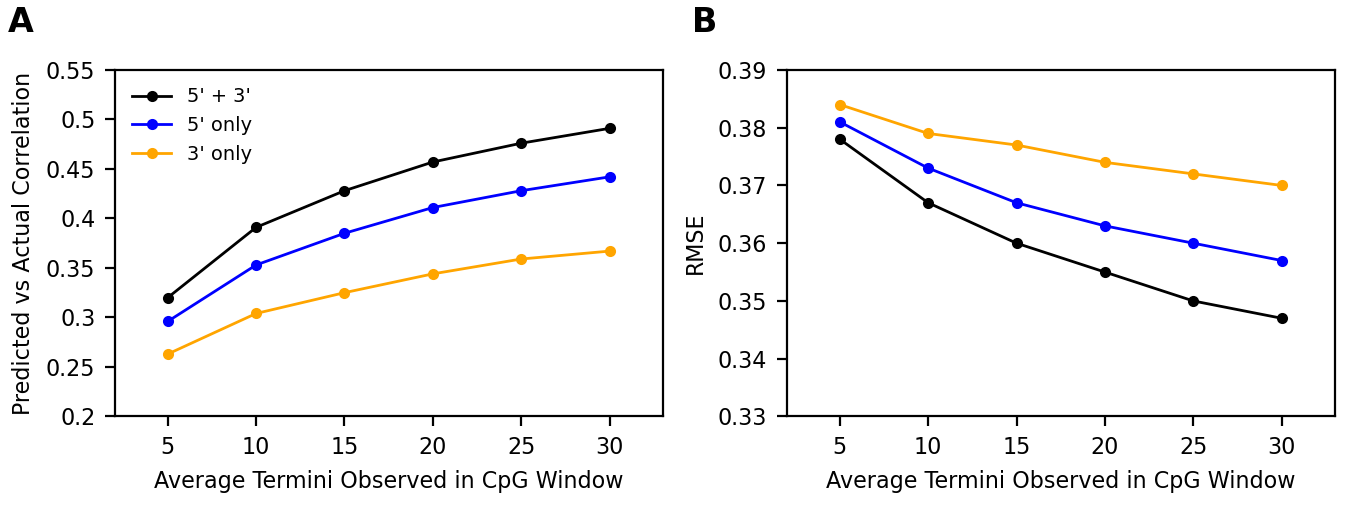


**Fig S6.** Effect of downsampling termini observances from plasma data on the performance of logistic regression models using 5’ and 3’ termini as features, 5’ only, and 3’ only. Pearson correlation between predicted and array beta values are shown left (A) and RMSE is shown right (B).
